# Neurogenic Rosacea and Facial Dysesthesia: Neurovascular–Immune Mechanisms and Translational Therapeutic Perspectives

**DOI:** 10.3390/medicina62091689

**Published:** 2026-09-03

**Authors:** Serap Maden

**Affiliations:** Department of Dermatology, Faculty of Medicine, Near East University, 99138 Nicosia, Cyprus; serap.maden@neu.edu.tr

**Keywords:** neurogenic rosacea, facial dysesthesia, sensory symptoms, neurogenic inflammation, mast cell activation, translational therapeutics

## Abstract

*Background and Objectives*: Neurogenic rosacea is a clinically useful, but not yet standardized, sensory-weighted presentation within the rosacea spectrum. This narrative review evaluates its clinical features, differential diagnosis, proposed mechanisms, and reported therapeutic approaches while distinguishing direct human evidence from indirect and experimental findings. *Materials and Methods*: PubMed/MEDLINE, Scopus, Web of Science, and Google Scholar were searched through 30 June 2026 using predefined combinations of terms related to rosacea, facial dysesthesia, neurogenic inflammation, sensory pathways, mast cells, and treatment. English-language clinical and experimental publications were selected by relevance; reference lists were also screened. Evidence was grouped as phenotype-specific human evidence, human evidence from rosacea populations or related conditions, case-based evidence, and animal or cellular evidence. *Results*: Direct evidence specific to neurogenic rosacea is limited mainly to small observational series and case reports. Human rosacea studies support neurovascular involvement, whereas many TRP-channel, protease, LL-37–MRGPRX2, and mast cell links remain indirect or experimental. No validated diagnostic criteria or phenotype-specific treatment algorithms exist. *Conclusions*: Neurogenic rosacea is best regarded as a clinically useful but not yet standardized sensory-weighted presentation within the rosacea spectrum, as current evidence does not establish it as an independent phenotype. The clinical features summarized in this review may support recognition of this presentation and its differentiation from relevant mimics, while diagnostic and treatment decisions should remain individualized. Prospective cohorts, validated sensory measures, biomarkers, and controlled phenotype-stratified trials are needed.

## 1. Introduction

Facial burning, stinging, dysesthesia, warmth, pruritus, flushing, and trigger-induced discomfort are not merely secondary complaints in rosacea; in many patients, they represent the main source of disease burden and quality-of-life impairment. Rosacea is a chronic inflammatory disorder of the centrofacial skin with heterogeneous vascular, inflammatory, ocular, and sensory manifestations. Although rosacea has traditionally been classified according to clinical subtypes, current phenotype-based approaches allow visible signs and patient-reported symptoms to be assessed together, which is particularly important when sensory symptoms are disproportionate to visible erythema or inflammatory lesions [1,2,3,4,5,6].

The pathogenesis of rosacea is multifactorial and cannot be explained solely by vascular dysfunction. Genetic susceptibility, environmental exposures, dysregulated innate and adaptive immune responses, microbial factors, barrier impairment, oxidative stress, and neurovascular dysfunction all contribute to disease expression [3,7,8,9,10]. Among these mechanisms, neurovascular and neuroimmune pathways have gained increasing attention because they may contribute to flushing, persistent erythema, temperature sensitivity, sensory discomfort, and trigger reactivity in rosacea [7,8,11].

Neurogenic rosacea was described as a symptom-dominant presentation characterized by facial burning, stinging, dysesthesia, heat sensation, pruritus, flushing, marked trigger reactivity, and incomplete response to conventional rosacea therapy [12]. Subsequent reports have used the terms neurogenic or neuropathic rosacea, but available evidence does not establish an independent phenotype with validated diagnostic boundaries [13,14,15]. In this review, the term is therefore used pragmatically to denote a clinically useful, but not yet standardized, sensory-weighted presentation within the rosacea spectrum.

A neurovascular–immune model is biologically plausible, but its components are supported by different levels of evidence. Current evidence in rosacea supports alterations in sensory and vascular signaling; however, many proposed links involving TRP channels, neuropeptides, LL-37, proteases, MRGPRX2, and mast cells are based on studies of general rosacea populations, other inflammatory disorders, animal models, or cellular systems [7,8,11,16,17,18].

This narrative review therefore addresses four questions: how a sensory-weighted presentation may be recognized without treating it as a validated diagnosis; how it can be distinguished from erythematotelangiectatic rosacea and important mimics; which mechanisms are supported by direct, indirect, or experimental evidence; and what is known about the efficacy, reported dosing, safety, and evidentiary limitations of therapeutic approaches.

## 2. Materials and Methods

This narrative review was conducted to synthesize clinical and mechanistic literature relevant to sensory-weighted rosacea. Searches were performed in PubMed/MEDLINE, Scopus, Web of Science, and Google Scholar from database inception to 30 June 2026. The core search sequence was as follows: (rosacea OR “neurogenic rosacea” OR “neuropathic rosacea”) AND (burning OR stinging OR dysesthesia OR “sensory symptoms” OR flushing OR “small fiber neuropathy” OR erythromelalgia). Mechanism-focused searches combined rosacea with (“neurogenic inflammation” OR “transient receptor potential” OR TRPV1 OR TRPA1 OR neuropeptide OR neuropeptides OR CGRP OR PACAP OR “mast cell” OR “mast cells” OR LL-37 OR MRGPRX2 OR protease OR proteases). Treatment-focused searches combined the disease terms with (treatment OR gabapentin OR pregabalin OR duloxetine OR hydroxychloroquine OR cromolyn OR brimonidine OR botulinum). English-language original human studies, case series, case reports, experimental studies, and clinically relevant reviews were eligible. Publications were excluded when they did not address rosacea-related sensory manifestations, a relevant differential diagnosis, a proposed pathway, or a reported intervention. Reference lists of included articles were screened for additional sources. Because this review was conducted as a narrative synthesis and no record-level screening log was maintained, counts of records identified, deduplicated, screened, and excluded were not available. Evidence was evaluated qualitatively and classified as direct phenotype-specific human evidence, human evidence from rosacea populations or related disorders, case-series or case-report evidence, and animal or cellular evidence. The literature search, study selection, and qualitative evidence assessment were performed by the author.

## 3. Findings of the Narrative Synthesis: Clinical Presentation and Proposed Diagnostic Framework

Neurogenic rosacea is not a validated independent subtype. For clinical discussion, it may be considered when a patient who otherwise meets phenotype-based rosacea criteria has persistent centrofacial burning, stinging, dysesthesia, warmth, or pruritus that is disproportionate to visible inflammatory activity; reproducible trigger sensitivity; and substantial symptom burden [5,6,12,13,14,15]. Flushing or persistent centrofacial erythema supports the association with rosacea, but neither sensory symptoms nor poor treatment response is specific. Papules and pustules may be absent or present [5,6,12,13,14,15]. The framework presented in Table 1 is intended to support clinical assessment and should not be interpreted as validated diagnostic criteria. Alternative dermatologic, neurologic, and vascular conditions should be considered, particularly when the presentation is atypical [1,6,14,15,19].

The main practical distinction from erythematotelangiectatic rosacea lies in the relative clinical weighting of features rather than in a proven biological boundary. In erythematotelangiectatic rosacea, transient or persistent erythema and telangiectasia usually define the dominant clinical burden; in the sensory-weighted presentation, persistent burning, stinging, dysesthesia, or pain is functionally important and often disproportionate to visible erythema [1,5,6,12,13,14,15]. This distinction is provisional, and substantial overlap is expected. Given this overlap, alternative diagnoses should be considered when symptoms are severe, unilateral, dermatomal, electrically triggered, or extend beyond the centrofacial region, particularly when flushing is accompanied by sweating or other systemic manifestations [14,15,19]. Phenotype-specific evidence is derived mainly from small clinical series, observational data, case-based reports, and narrative analyses [12,13,14,15].

Human provocation and biomarker studies in unselected rosacea populations support neurovascular involvement, including PACAP-induced flushing and edema, as well as elevated circulating CGRP levels [20,21], but do not establish that these abnormalities are unique to neurogenic rosacea. Table 1 therefore separates clinical observations from indirect human and preclinical experimental evidence.

## 4. Hierarchy of Evidence for Proposed Neurovascular–Immune Pathways

### 4.1. Direct and Indirect Human Evidence

Phenotype-specific human mechanistic evidence is sparse. Available clinical reports describe the sensory presentation but do not define biomarkers or establish causality [12,13,14,15]. In rosacea populations not selected for a sensory-weighted presentation, histologic and molecular studies have reported altered innervation and TRP-channel expression, while clinical studies have documented PACAP-provoked flushing and facial edema and elevated plasma CGRP levels [20,21,22,23]. These observations support neurovascular involvement in rosacea generally, but extrapolation to a distinct neurogenic phenotype remains indirect.

### 4.2. Animal and Cellular Evidence

**Figure 1:**
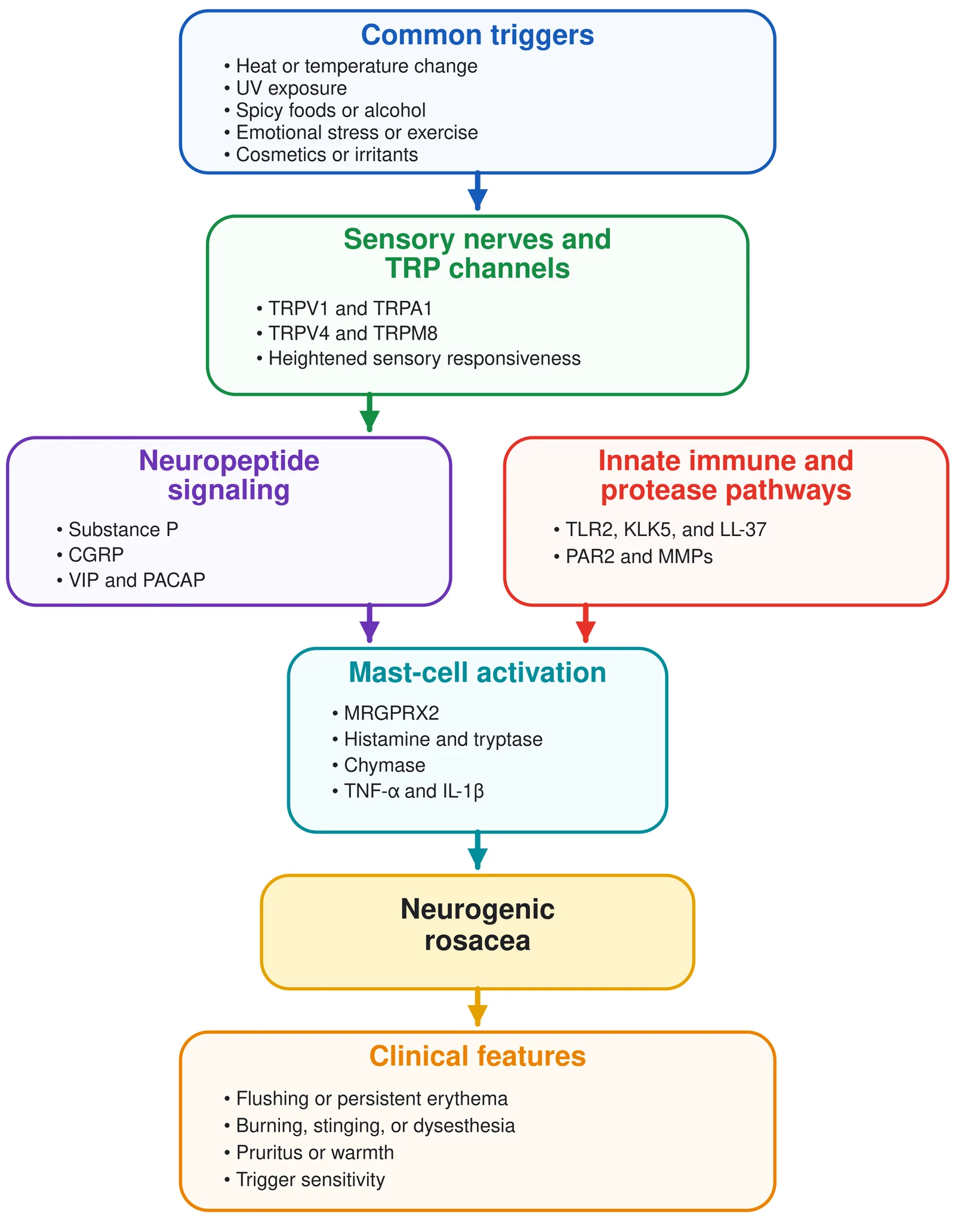
Proposed neurovascular–immune pathways associated with the clinical features of neurogenic rosacea. Observed clinical features, including triggers, flushing, erythema, and sensory symptoms, are based on clinical reports. Neurovascular findings are supported indirectly by human studies in rosacea populations. Links involving LL-37, proteases, MRGPRX2/MrgprB2, mast cells, and inflammatory amplification are derived predominantly from animal or cellular models. Arrows denote hypothesized relationships rather than confirmed phenotype-specific causal links. CGRP, calcitonin gene-related peptide; IL-1β, interleukin-1 beta; KLK5, kallikrein 5; LL-37, cathelicidin antimicrobial peptide LL-37; MMPs, matrix metalloproteinases; MRGPRX2, Mas-related G protein-coupled receptor X2; PACAP, pituitary adenylate cyclase-activating polypeptide; PAR2, protease-activated receptor 2; TLR2, Toll-like receptor 2; TNF-α, tumor necrosis factor alpha; TRP, transient receptor potential; UV, ultraviolet; VIP, vasoactive intestinal peptide.

Rosacea-like mouse models and in vitro studies link sensory neurons, TRP signaling, LL-37, proteases, mast cell activation, and inflammatory amplification [24,25,26,27,28,29,30,31,32,33]. Such studies provide preliminary biological support; however, they cannot establish that the same pathway is necessary, sufficient, or selectively enriched in patients with a sensory-weighted presentation. Figure 1 summarizes these proposed relationships, with the sources of supporting evidence specified in the figure legend.

In the skin, TRP channels form a broad sensory-signaling network rather than a pathway confined to peripheral nerves alone. Although they are expressed on sensory nerve terminals, their distribution also includes keratinocytes, endothelial cells, vascular smooth muscle cells, and several immune cell populations [34,35]. The expression of TRP channels in neural, vascular, epithelial, and immune compartments supports their possible involvement in rosacea-related trigger sensitivity, neurovascular reactivity, inflammatory signaling, and sensory symptoms such as burning, stinging, and cutaneous discomfort [22,23,36].

The potential involvement of TRP-dependent pathways in rosacea is supported by their capacity to stimulate the release of vasoactive and proinflammatory neuropeptides, including substance P, CGRP, VIP, and PACAP. These mediators may contribute to vasodilation, edema, pain, pruritus, and inflammatory amplification [18,36,37]. Histologic and molecular studies further support neural participation in rosacea. Erythematotelangiectatic rosacea has been associated with altered sensory innervation, and increased expression of TRP-related channels has been detected in neural, vascular, and immune compartments of rosacea-affected skin. Together, these findings are consistent with heightened neurovascular responsiveness in rosacea, whereby common stimuli may elicit flushing and sensory symptoms [22,23,36].

### 4.3. TRP Channels and Trigger Sensitivity

Among TRP channel subtypes, activation profiles of transient receptor potential vanilloid 1 (TRPV1) and transient receptor potential ankyrin 1 (TRPA1) correspond closely to several thermal, chemical, dietary, and environmental triggers commonly implicated in rosacea exacerbation. TRPV1 is activated by noxious heat, capsaicin, ethanol, and inflammatory mediators, whereas TRPA1 responds to cold exposure and irritant chemicals, including formalin/formaldehyde-related compounds [38,39,40]. Other TRP channels may also contribute to trigger sensitivity. Transient receptor potential vanilloid 4 (TRPV4) can respond to heat, osmotic changes, and inflammatory metabolites, while transient receptor potential melastatin 8 (TRPM8) is activated by cold and menthol [23,41].

Dietary and environmental factors provide clinically recognizable correlates of these proposed pathways. Spicy foods, alcohol, heat, sun exposure, and stress are commonly recognized as patient-specific triggers of rosacea [7,42,43]. Capsaicin, derived from chili peppers, is a TRPV1 agonist [38]; cinnamaldehyde-containing foods may activate TRPA1-associated pathways [42]; and formalin/formaldehyde-related compounds can stimulate TRPA1-dependent nociceptive signaling under experimental conditions [44].

TRPV1 and TRPA1 activation may further propagate neurogenic inflammation by stimulating substance P and CGRP release. The altered expression and sensitization of sensory pathways described in rosacea provide a potential explanation for enhanced responsiveness to thermal and chemical stimuli. However, a consistently reduced sensory activation threshold has not been established specifically for neurogenic rosacea [18,23,24,36,45,46,47]. TRP signaling may also interact with prostaglandin E2 pathways. Such reciprocal sensitization can reduce the activation threshold of sensory channels and intensify pain perception, thereby linking inflammatory mediators to heightened sensory responses [48,49].

Increased expression of several TRPV channels, including TRPV1, TRPV2, TRPV3, and TRPV4, has been reported in rosacea-affected skin [23]. Experimental models have also linked LL-37 to TRPV4 upregulation and mast cell degranulation, suggesting that this peptide may amplify rosacea-like inflammation through interactions among innate immune, neurovascular, and mast cell pathways [25,50].

### 4.4. Neuropeptides, Vasodilation, and Sensory Symptoms

Neuropeptide signaling offers a plausible mechanism through which neural activation may be translated into vascular and sensory manifestations in rosacea. Substance P may contribute to neurogenic edema and inflammatory amplification, in part through neurokinin 1 (NK1) receptor-mediated effects on postcapillary venules and mast cells. CGRP, VIP, and PACAP are strongly vasodilatory mediators and can promote vascular relaxation through actions on vascular smooth muscle cells and endothelial pathways [51,52]. PACAP may also be released from autonomic nerve fibers and may enhance nitric oxide production by endothelial cells, thereby further supporting vasodilatory responses [52].

Among these mediators, CGRP is primarily associated with arteriolar vasodilation, whereas substance P may promote plasma extravasation, edema, nociceptive signaling, and mast cell activation [36,51,52]. Preliminary clinical evidence comes from a small, open-label, nonrandomized trial in which erenumab was associated with fewer days of moderate-to-severe flushing and erythema in patients with rosacea [53]. Evidence linking neuropeptide signaling to persistent sensory symptoms remains more limited; however, these mediators may contribute to facial warmth, dysesthesia, and altered cutaneous sensitivity [18,22].

The contribution of TRP-channel activation to rosacea is not limited to vascular reactivity. By promoting neuropeptide release from sensory nerves, TRP-dependent signaling may also influence immune cell recruitment, mast cell activation, and inflammatory amplification [18,36]. Mast cells are particularly relevant in this context because they can respond to neuropeptides and release histamine, tryptase, cytokines, chemokines, and matrix metalloproteinases, all of which may reinforce vascular changes, sensory nerve activation, and local inflammation [16,37,54]. In addition, mast cell-derived proteases and matrix-remodeling mediators may contribute to stromal changes and tissue remodeling, suggesting that neurogenic inflammation may interact with structural alterations in rosacea skin rather than acting solely as an acute vascular reflex [17,22].

Substance P is also relevant within this neuroimmune network because it can modulate local blood flow and directly activate mast cells [37,54]. In human mast cells, substance P has been shown to induce pro-inflammatory cytokines such as tumor necrosis factor alpha (TNF-α) and interleukin (IL)-3, as well as chemokines including C-C motif chemokine ligand (CCL) 2, C-X-C motif chemokine ligand (CXCL) 9, CXCL10, CCL5, and CXCL8/IL-8, supporting a role for neuropeptide-driven immune activation in rosacea pathophysiology [54,55]. Neuropeptides may further sustain inflammation by promoting IL-1β expression and enhancing leukocyte recruitment through increased vascular adhesion molecule expression [56,57].

Thus, neurogenic inflammation in rosacea may be considered not only as a transient vascular response but also as a process linked to immune cell recruitment, inflammatory amplification, and persistent sensory symptoms [18,58]. Neuroimmune crosstalk may additionally involve keratinocytes, fibroblasts, endothelial cells, and mast cells, which can interact with sensory nerves through cytokines, inflammatory mediators, and neuropeptides [17,59]. Sensory neurons may also participate in innate immune signaling through the expression of pattern-recognition receptors, including Toll-like receptors, thereby providing a potential interface between microbial or damage-associated signals and neural activation [23,60].

### 4.5. Protease-Dependent Innate Immune Amplification

Protease-dependent mechanisms may provide an additional bridge between epidermal barrier dysfunction, innate immune activation, and neurogenic inflammation. Matriptase and caspase-14 participate in filaggrin processing and epidermal barrier integrity, while kallikrein 5 (KLK5) and kallikrein 7 (KLK7) contribute to physiological desquamation. In rosacea, increased KLK5 expression, partly related to enhanced Toll-like receptor 2 (TLR2) signaling, may promote excessive cathelicidin processing and increase the generation of bioactive peptide fragments with vasodilatory and chemotactic properties [26,61,62,63].

Enhanced serine protease activity may also influence sensory signaling through protease-activated receptors. Cross-sensitization between protease-activated receptor (PAR) 2 and TRP pathways provides a plausible mechanism by which protease activity may intensify neurogenic inflammation, pain signaling, and cutaneous hypersensitivity in rosacea [64,65]. This protease activity is consistent with an innate immune amplification loop in which TLR2 upregulation increases epidermal serine protease production and downstream inflammatory responses [26]. Reduced endogenous protease inhibition may further shift the balance toward protease-driven cutaneous activation [66].

Furthermore, proteases can modulate vasodilation, inflammation, pain, pruritus, and immune function through both PAR-dependent and PAR-independent mechanisms [67,68]. Most evidence supporting the proposed barrier–protease–PAR–TRP interaction is derived from experimental skin biology and inflammatory pain models rather than from phenotype-specific human studies of rosacea [61,62,64,65,66,67,68]. Accordingly, the potential relevance of this pathway to sensory-weighted rosacea remains to be confirmed in clinical studies.

Mast cells may act as amplifiers of inflammation in rosacea because they are located near vessels and sensory nerves and can respond to neuropeptides and innate immune signals [16,37,69]. In human mast cells, LL-37 can activate MRGPRX2, and LL-37-induced rosacea-like inflammation is attenuated in mast cell-deficient experimental models [27,28,29,30]. Additional animal studies involving MRGPRX2/MrgprB2 and TRPV4 support non-IgE-mediated mast cell activation [25,31,32,33].

These findings are predominantly derived from cellular or animal studies [25,27,28,29,30,31,32,33]. Available phenotype-specific clinical reports do not demonstrate that mast cell number, activation, or LL-37–MRGPRX2 signaling is selectively increased in neurogenic rosacea compared with other rosacea presentations [12,13,14,15]. Mast cells should therefore be regarded as a potential interface between innate immune and neural pathways rather than as an established central driver of the sensory-weighted presentation.

## 5. Differential Diagnosis

Burning, flushing, warmth, and facial dysesthesia may occur across several dermatologic, neurologic, and systemic conditions. Table 2 compares this presentation with its principal differential diagnoses. The need for additional investigations depends on the clinical history and examination. Patch testing may be useful when the morphology or exposure history suggests allergic contact dermatitis. Neurologic assessment, including evaluation for small-fiber neuropathy, may be considered in patients with distal symptoms, objective sensory abnormalities, or autonomic features. Endocrine evaluation may be warranted when flushing is atypical or accompanied by systemic manifestations.

## 6. Management

Management should begin with confirmation of the rosacea phenotype, gentle skin care, photoprotection, trigger modification, and treatment of established visible manifestations. Persistent sensory symptoms should be measured separately from erythema or lesion counts. The following approaches reflect treatments described in the available literature rather than a validated management algorithm. Their reported efficacy should be interpreted cautiously because the evidence is derived mainly from small, heterogeneous, and uncontrolled studies [4,12,13,15].

Conventional rosacea-directed therapies have established roles in the management of inflammatory lesions and persistent erythema, although improvement in visible signs may not be accompanied by improvement in burning or dysesthesia [4,12,13,15]. Neuromodulators have been evaluated primarily in small series, observational studies, and case-based reports [12,13,15]. 

No comparative trial has established the superiority of gabapentin, pregabalin, duloxetine, tricyclic antidepressants, botulinum toxin, cromolyn, hydroxychloroquine, or combination therapy specifically for neurogenic rosacea. Off-label systemic treatment requires individualized risk assessment, slow titration when appropriate, review of comorbidities and interactions, and monitoring according to the agent used. Laser and light-based procedures are established options for selected vascular manifestations of rosacea, but evidence specific to a sensory-weighted presentation remains indirect [4,74].

**Figure 2:**
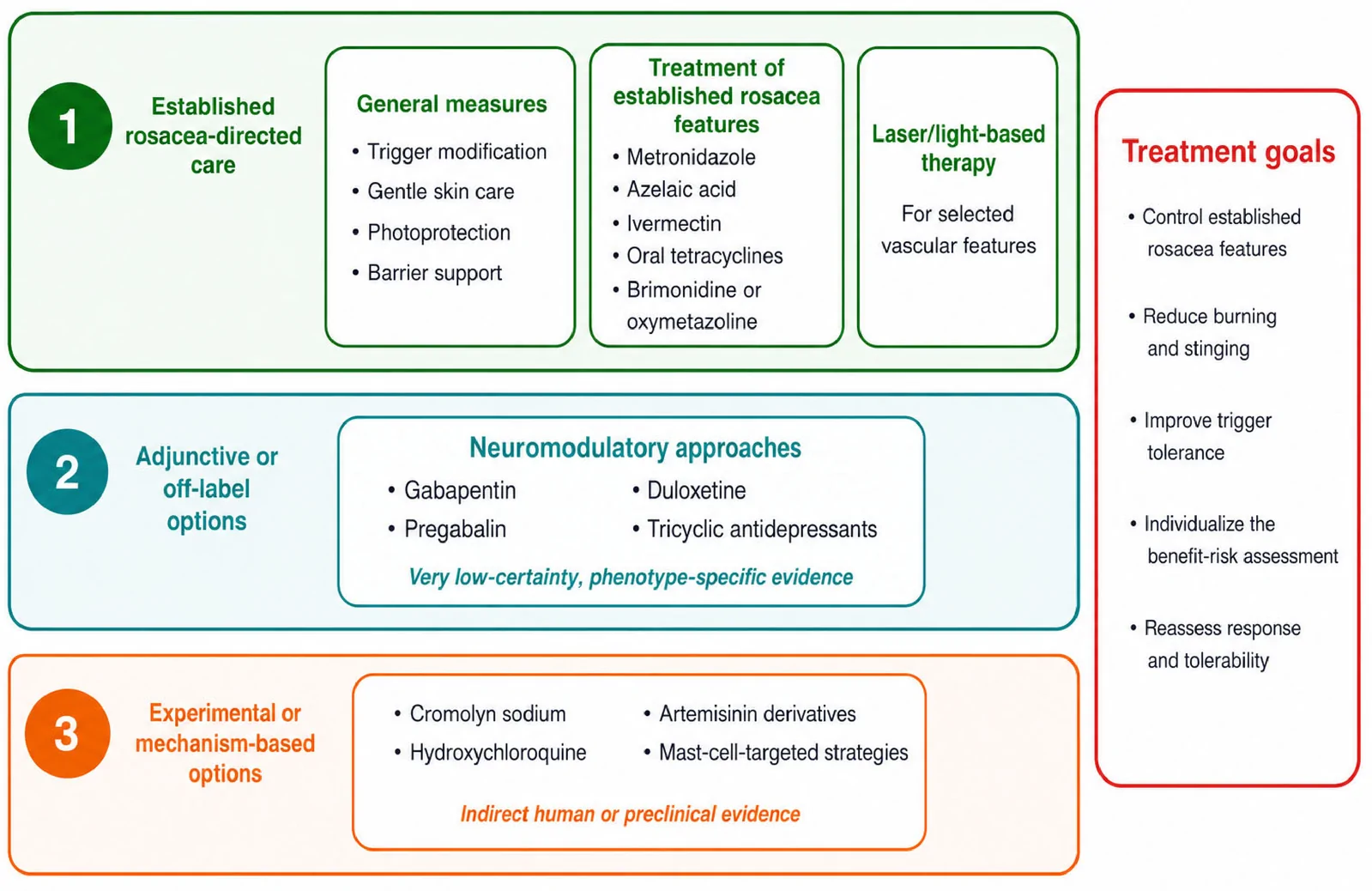
Therapeutic approaches in neurogenic rosacea. The figure separates established rosacea-directed care from adjunctive/off-label neuromodulatory options and experimental or mechanism-based approaches. The categories indicate evidence status and do not imply regulatory approval, equivalent efficacy, or a treatment sequence.

Figure 2 summarizes management domains according to the dominant clinical burden and distinguishes established, adjunctive, and experimental approaches by evidence status.

The reported regimens, outcomes, safety considerations, and levels of evidence are summarized in Table 3.

Mast cell-directed and other pathway-targeted agents are supported mainly by studies in rosacea populations or by experimental models [30,76,77,78,79,80,81,82].

## 7. Discussion

The principal contribution of this review is to integrate the clinical features of a sensory-weighted rosacea presentation with the available neurovascular, immune, and therapeutic evidence. Although this presentation is clinically recognizable, its diagnostic boundaries and phenotype-specific evidence remain incompletely defined.

Sensory-weighted facial burning, stinging, dysesthesia, warmth, pruritus, and trigger sensitivity are clinically important, but current publications do not confirm an independent neurogenic rosacea phenotype or define validated diagnostic thresholds [12,13,14,15]. The proposed framework is therefore descriptive and exclusion-based.

The strongest phenotype-specific evidence remains clinical and case-based. Human studies in rosacea populations support neurovascular dysregulation, while the proposed TRP, neuropeptide, protease, LL-37–MRGPRX2, and mast cell network relies substantially on indirect human observations and animal or cellular models [20,21,22,23,24,25,26,27,28,29,30,31,32,33]. This hierarchy prevents biological plausibility from being mistaken for demonstrated phenotype-specific causality.

Mast cells are one candidate amplifier rather than an established defining mechanism. Their role is supported by experimental rosacea-like inflammation, but no comparative human study has demonstrated selective mast cell activation in sensory-weighted disease [16,30,31,32,33].

Therapeutic evidence is similarly limited. Standard rosacea therapy should address established visible manifestations, whereas neuromodulators and mechanism-based approaches remain off-label, adjunctive, or investigational for sensory-weighted disease. Published doses and outcomes are heterogeneous, and no treatment can currently be recommended as an evidence-based phenotype-specific standard.

## 8. Limitations and Future Directions

This review is limited by its narrative design and heterogeneity of terminology. The restriction to English-language publications, reliance on selected databases, and relevance-based source selection may have resulted in the omission of pertinent studies. Publication bias cannot be excluded. In addition, the literature search, study selection, and qualitative evidence assessment were performed by a single author and may therefore have been influenced by subjective judgment. Most direct clinical evidence comprises small observational studies, case series, and case reports, whereas evidence concerning the proposed biological pathways is derived largely from rosacea populations not selected for a sensory-weighted presentation, related disorders, or experimental models. Future studies should establish consensus terminology; validate sensory and quality-of-life measures; compare sensory-weighted presentations with erythematotelangiectatic rosacea and relevant mimics; and evaluate biomarkers and treatments in prospectively characterized cohorts. Controlled trials should report clinician-assessed signs and patient-reported burning, stinging, dysesthesia, trigger sensitivity, tolerability, and quality of life separately. Objective biomarkers and noninvasive measures of neurovascular activity are additional priorities. A systematic review identified candidate inflammatory biomarkers in rosacea, including IL-1β, TNF-α, IL-37, IFN-γ, and MMP-9, but none has been validated for identifying a sensory-weighted presentation [83]. Dynamic optical coherence tomography (D-OCT) can visualize and quantify in vivo cutaneous microvascular morphology and has detected vascular changes after topical brimonidine in rosacea [84]. Quantitative imaging could therefore complement clinician-rated erythema and patient-reported sensory outcomes in future phenotype-stratified studies. D-OCT monitoring of vascular remodeling during therapy in other dermatoses illustrates a potential translational model, although direct applicability to neurogenic rosacea remains hypothetical [85].

## 9. Conclusions

Neurogenic rosacea should be regarded as a clinically useful, but not yet standardized, sensory-weighted presentation within the rosacea spectrum—not as a confirmed independent phenotype. Recognition may improve assessment of symptom burden and prompt targeted evaluation for dermatologic, neurologic, vascular, menopausal, medication-related, and endocrine mimics.

Phenotype-specific human evidence remains limited but continues to evolve, while several proposed neurovascular–immune pathways are informed partly by indirect and experimental findings. Neuromodulatory and pathway-directed approaches may have a role in selected patients, with appropriate consideration of dosing, safety, and the strength of the available evidence. Further studies should refine clinical characterization, evaluate candidate biomarkers, and contribute to the development of evidence-based diagnostic and treatment strategies.

## Figures and Tables

**Table 1:** Clinical features that may support recognition of neurogenic rosacea.

Clinical Feature	Suggested Features	Qualification
Rosacea context	Centrofacial flushing, persistent erythema, telangiectasia, and/or other compatible rosacea manifestations.	Sensory symptoms alone are insufficient to establish an association with rosacea.
Prominent sensory burden	Persistent or recurrent burning, stinging, dysesthesia, warmth, pruritus, or pain that is clinically important and may be disproportionate to visible inflammation.	No validated symptom threshold or phenotype-specific diagnostic criterion is available.
Supportive observations	Reproducible thermal, environmental, dietary, emotional, or cosmetic triggers; persistent sensory symptoms despite improvement of visible signs; treatment intolerance.	These observations are supportive but nonspecific, and overlap with erythematotelangiectatic rosacea is expected.
Features warranting consideration of alternative diagnoses	Markedly unilateral or dermatomal symptoms; electric-shock-like attacks; generalized or sweating-associated flushing; systemic, neurologic, or eczematous features.	Targeted evaluation should be guided by the clinical history and examination; routine extensive testing is not required in a typical presentation.

The clinical features summarized in this table are derived from published rosacea criteria and clinical descriptions [5,6,12,13,14,15]; sensitivity, specificity, and diagnostic validation data are not available.

**Table 2:** Concise differential diagnosis of sensory-weighted facial erythema and dysesthesia.

Condition	Distinguishing Clinical Features	Suggested Clinical Evaluation
Erythematotelangiectatic rosacea	Erythema/telangiectasia dominates; burning or stinging is generally less dominant.	Phenotype-based rosacea assessment; recognize overlap [1,5,6].
Facial erythromelalgia	Severe episodic burning, warmth, and erythema; frequent heat exacerbation and reported relief with cooling.	History during attacks; examine extremities; evaluate secondary causes when indicated [19].
Small-fiber neuropathy	Neuropathic pain, allodynia, altered pinprick/temperature sensation, distal symptoms, or autonomic complaints.	Neurologic examination; skin biopsy or autonomic testing in selected cases; neurogenic rosacea itself is not proven to be SFN [14].
Allergic/irritant contact dermatitis	Pruritus, scaling, fissuring, vesiculation, a distribution corresponding to exposure, new cosmetics/topicals, occupational exposure.	Exposure withdrawal; patch testing for suspected allergy [70].
Sensitive skin syndrome	Stinging/burning triggered by products or environment, often with minimal objective signs and without clear accompanying features of rosacea.	Structured exposure and skincare history; exclude dermatoses [71].
Trigeminal neuropathic pain	Unilateral or trigeminally distributed pain; trigeminal neuralgia is characterized by recurrent, brief, triggerable electric-shock-like attacks.	Focused neurologic examination; consider imaging or specialist referral when atypical neurologic features are present [72].
Menopausal flushing	Episodic hot flashes with sweating, upper-body spread, sleep disturbance, and perimenopausal timing.	Review menstrual and menopausal history; investigate further only when clinically indicated [73].
Endocrine/systemic flushing	Generalized or wet/dry episodic flushing with diarrhea, wheeze, palpitations, labile blood pressure, weight loss, fever, or medication temporal relationship.	Medication review and targeted testing/referral; avoid indiscriminate screening [73].

Clinical features are summarized to facilitate comparison among the principal differential diagnoses. Evaluation should be individualized according to the history, examination, exposure pattern, neurologic findings, and associated systemic symptoms. SFN, small-fiber neuropathy.

**Table 3:** Reported interventions, dosing, safety, and level of evidence.

Intervention	Reported Regimen/Target	Reported Outcome	Key Safety Considerations	Evidence Level
Gentle skincare, photoprotection, trigger modification	Individualized; no NR-specific regimen	May reduce irritant burden and supports standard rosacea care	Generally low risk; avoid over-restriction	Consensus-based rosacea care; no NR-specific trial [4]
Conventional topical/oral rosacea therapy	Label- or guideline-directed regimen for papules/pustules or erythema	Treats visible rosacea features; sensory response uncertain	Agent-specific irritation; antimicrobial stewardship and pregnancy-related precautions	Established for rosacea; indirect for NR [4]
Gabapentin	Variable off-label titration in reports; no standardized NR dose	Partial improvement in burning/flushing in small series	Somnolence, dizziness, edema; renal dose adjustment; taper gradually	Case series/observational reports [12,13,15]
Pregabalin ± duloxetine	Pregabalin 150 mg/day plus duloxetine 90 mg/day for 6 months in one case report	Marked reduction in pain and burning in the reported patient	Sedation/dizziness/edema; renal adjustment; duloxetine interactions and withdrawal precautions	Case-based evidence summarized in a clinical review; very low certainty [13]
Tricyclic antidepressant or duloxetine	Variable off-label dosing; no standardized NR regimen	Reported benefit in subsets of small series	Anticholinergic/cardiac effects for TCAs; nausea, blood pressure and interaction concerns for duloxetine	Case series/case reports [12,13]
Brimonidine 0.33% gel	Once-daily topical use for persistent erythema	Reduces erythema; effect on dysesthesia unproven	Paradoxical or rebound erythema; irritation	Established rosacea evidence; indirect for sensory symptoms [4,75].
Topical cromolyn sodium	4% topical formulation reported	Preliminary reduction in erythema	Local irritation; limited formulation and efficacy data	Small preliminary human evidence plus experimental data [30,76]
Hydroxychloroquine	Regimens evaluated in clinical rosacea studies; not standardized for NR	Pilot rosacea study suggested clinical improvement	Retinal, cardiac, hematologic and drug-interaction risks; monitoring required	Pilot randomized evidence in rosacea populations; indirect for NR [77,78]
Artemether/artemisinin derivatives	Topical artemether emulsion studied in papulopustular rosacea	Pilot improvement in inflammatory rosacea	Product-specific safety; insufficient long-term and NR data	Pilot study in rosacea populations plus experimental evidence [79,80]
Botulinum toxin A	Injection techniques and doses vary across reports	Possible improvement in refractory flushing/erythema	Pain, bruising, asymmetry, unwanted muscle weakness; procedural expertise required	Case-based clinical observations summarized in a review, together with predominantly experimental mechanistic evidence [13,81,82]

NR, neurogenic rosacea.

## Data Availability

No new data were created or analyzed in this study. Data sharing is not applicable to this article.

## Abbreviations

The following abbreviations are used in this manuscript:

CCL	C-C motif chemokine ligand
CGRP	calcitonin gene-related peptide
CXCL	C-X-C motif chemokine ligand
IL	interleukin
IgE	immunoglobulin E
IPL	intense pulsed light
KLK	kallikrein
KLK5	kallikrein 5
KLK7	kallikrein 7
LL-37	cathelicidin antimicrobial peptide LL-37
MMPs	matrix metalloproteinases
MMP-9	matrix metalloproteinase 9
MRGPRX2	Mas-related G protein-coupled receptor X2
MrgprB2	Mas-related G protein-coupled receptor B2
NK1	neurokinin 1
PACAP	pituitary adenylate cyclase-activating polypeptide
PAR2	protease-activated receptor 2
SP	substance P
TLR2	Toll-like receptor 2
TNF-α	tumor necrosis factor alpha
TRP	transient receptor potential
TRPA1	transient receptor potential ankyrin 1
TRPM8	transient receptor potential melastatin 8
TRPV1	transient receptor potential vanilloid 1
TRPV2	transient receptor potential vanilloid 2
TRPV3	transient receptor potential vanilloid 3
TRPV4	transient receptor potential vanilloid 4
UV	ultraviolet
VIP	vasoactive intestinal peptide
